# HABITAT: A longitudinal multilevel study of physical activity change in mid-aged adults

**DOI:** 10.1186/1471-2458-9-76

**Published:** 2009-03-05

**Authors:** Nicola W Burton, Michele Haynes, Lee-Ann M Wilson, Billie Giles-Corti, Brian F Oldenburg, Wendy J Brown, Katrina Giskes, Gavin Turrell

**Affiliations:** 1 The University of Queensland School of Human Movement Studies, Brisbane, Australia; 2 The University of Queensland School of Social Science, Brisbane, Australia; 3 Queensland University of Technology School of Public Health, Brisbane, Australia; 4 The University of Western Australia, Centre for the Built Environment and Health, School of Population Health, Perth, Australia; 5 Monash University Department of Epidemiology & Preventive Medicine, Melbourne, Australia

## Abstract

**Background:**

Little is known about the patterns and influences of physical activity change in mid-aged adults. This study describes the design, sampling, data collection, and analytical plan of HABITAT, an innovative study of (i) physical activity change over five years (2007–2011) in adults aged 40–65 years at baseline, and (ii) the relative contribution of psychological variables, social support, neighborhood perceptions, area-level factors, and sociodemographic characteristics to physical activity change.

**Methods/Design:**

HABITAT is a longitudinal multi-level study. 1625 Census Collection Districts (CCDs) in Brisbane, Australia were ranked by their index of relative socioeconomic disadvantage score, categorized into deciles, and 20 CCDs from each decile were selected to provide 200 local areas for study inclusion. From each of the 200 CCDs, dwellings with individuals aged between 40–65 years (in 2007) were identified using electoral roll data, and approximately 85 people per CCD were selected to participate (N = 17,000). A comprehensive Geographic Information System (GIS) database has been compiled with area-level information on public transport networks, footpaths, topography, traffic volume, street lights, tree coverage, parks, public services, and recreational facilities Participants are mailed a questionnaire every two years (2007, 2009, 2011), with items assessing physical activity (general walking, moderate activity, vigorous activity, walking for transport, cycling for transport, recreational activities), sitting time, perceptions of neighborhood characteristics (traffic, pleasant surroundings, streets, footpaths, crime and safety, distance to recreational and business facilities), social support, social cohesion, activity-related cognitions (attitudes, efficacy, barriers, motivation), health, and sociodemographic characteristics. Analyses will use binary and multinomial logit regression models, as well as generalized linear latent growth models.

**Discussion:**

HABITAT will provide unique information to improve our understanding of the determinants of physical activity, and to help identify "people" and "place" priority targets for public policy and health promotion aimed at increasing physical activity participation among mid-aged men and women.

## Background

To promote and maintain health, it is recommended that adults do moderate intensity exercise for at least 30 minutes on five days each week, or vigorous intensity activity for at least 20 minutes on three days each week, or a combination of these [1]. Mid-aged Australians (45–59 years), and in particular men, are least likely to achieve these recommendations, with one in every two people insufficiently active [2]. Studies from other countries have also demonstrated lower rates of activity in mid-aged adults compared with younger cohorts [3,4].

Understanding the factors enabling and limiting physical activity in mid-age is therefore, a priority to identify potential mechanisms of change for promotion strategies. Social ecological theory posits that the multi-level determinants of behaviour include psychological, social, and environmental factors [5,6], and other authors have reviewed the associations between such factors and physical activity [7-11]. Traditionally, research has focused on psychological influences such as enjoyment, attitudes, efficacy, outcome expectancies (e.g., improved appearance, weight management, social interactions), and perceived barriers (e.g., work/family commitments, fatigue, disinterest). Social factors involve social cohesion, and support from significant others such as a partner, peers, and health professionals. Recently, there is increasing interest in environmental factors, such as safety, aesthetics, the availability of facilities, population density, distance to facilities, street connectivity, land use, and traffic. Sociodemographic influences include gender, health, and socioeconomic position. Life events, such as marriage, having children, and retirement, may also influence activity levels.

Many of these factors are measured at the level of the individual, which typically involves surveys of subjective perceptions and experiences. Area-level measurement involves more objective assessment, such as census data or environmental audits. Environmental influences, for example, can be measured as perceptions of the availability of facilities (individual-level), or an actual count of facilities (area-level). Socioeconomic factors can be measured as self-reported income or education (individual-level) or via census-based measures of neighborhood disadvantage (area-level). Multi-level studies are therefore needed to determine the influence of area-level factors above and beyond that of individual-level factors: To what extent are differences in physical activity because of the areas people live in (i.e. contextual effects) or because of the people living in those areas (i.e. compositional effects)?

Cross sectional studies of the associations between these influences and physical activity are limited by an inability to determine causality, and assume that influences are stable over time. Repeated cross sectional studies of activity prevalence may not reveal the extent of underlying patterns of change because of a cancellation of effects. Data from the Australian Longitudinal Study on Women's Health, for example, indicate that while the *overall *proportion of active mid-aged women increased by 9% between 2001 and 2004, 26% of women categorized as inactive in 2001 were active in 2004, and 18% who were active became inactive [12]. Longitudinal cohort studies are needed therefore, to determine the patterns of adoption, maintenance, and discontinuation of activity, and the factors associated with these changes.

Minimal work has been done on empirically contrasting activity influences using the same sample [13-15]. Studies that focus on only one type of influence are not optimizing our ability to assess the maximal amount of variation in activity. Focusing on only one type of influence may over-estimate the amount of unique variance accounted for, and lead to erroneous conclusions of strength of an association. There is a need therefore, to conduct more research that integrates psychological, social, and environmental variables [16]. This approach is more likely to reflect the many factors associated with activity and the varied opportunities for intervention [16,17]. Studies incorporating multiple influences will also facilitate the comparison of their relative importance, so as to identify priorities for change strategies. De Bourdeaudhuij [18] for example, reported that social variables may provide the most unique explanatory power about activity participation. Giles-Corti and Donovan [19] however, reported that activity was more strongly associated with individual-level variables, such as cognition, than either social or physical environmental variables.

Many studies have conceptualized physical activity as a homogenous behavior, making the assumption that influences are similarly associated with the different domains of activity [16]. There is evidence however, to suggest that this is not the case. Sallis and colleagues reported that self efficacy was associated with adopting vigorous activity, while health knowledge was associated with adopting moderate activity [20]. Humpel and colleagues found that different environmental attributes were associated with general neighborhood walking, walking for exercise, walking for pleasure, and walking to get to and from places [21]. Our previous research indicated that physical health, discouragement, and time management accounted for more unique variation in vigorous activity; anticipated social interactions and weight management contributed more to moderate-intensity activity; and neighborhood aesthetics contributed more to walking [22]. Accordingly, there is a need for studies that differentiate among the domains of physical activity.

### Study Aims

HABITAT (How Areas in Brisbane Influence HealTh and AcTivity) is a longitudinal multi level study of physical activity in mid-aged men and women, and examines the relative contribution of psychological, social, perceived environmental, area-level, and sociodemographic factors. The study aims to

1. Assess the patterns (directions and magnitude) of physical activity change between 2007 and 2011 in men and women aged 40–65 years (in 2007).

2. Examine the relative contributions of psychological, social, environmental, area level, and sociodemographic factors to change in physical activity.

3. Examine the associations of psychological, social, environmental, area level, and sociodemographic factors with different types of activity, including general walking, walking for transport, cycling for transport, moderate intensity activity, and vigorous intensity activity.

## Methods/Design

### Funding and Support

This project was awarded funding by the (Australian) National Health and Medical Research Committee (ID290521; ID497236). Brisbane City Council provided additional funding support and specific geographical data.

### Ethical Clearance

This project was awarded ethical clearance by The University Human Research Ethics Committee at the Queensland University of Technology (ID3967H).

### Study Design

HABITAT is a longitudinal multi level study (2007–2011).

### Setting

HABITAT is being conducted in the Statistical Subdivision of Brisbane in Australia. Brisbane is the capital city of the state of Queensland, and the third largest city in Australia with a population of approximately 1.8 million.

### Study Areas and Participants

**Figure 1:**
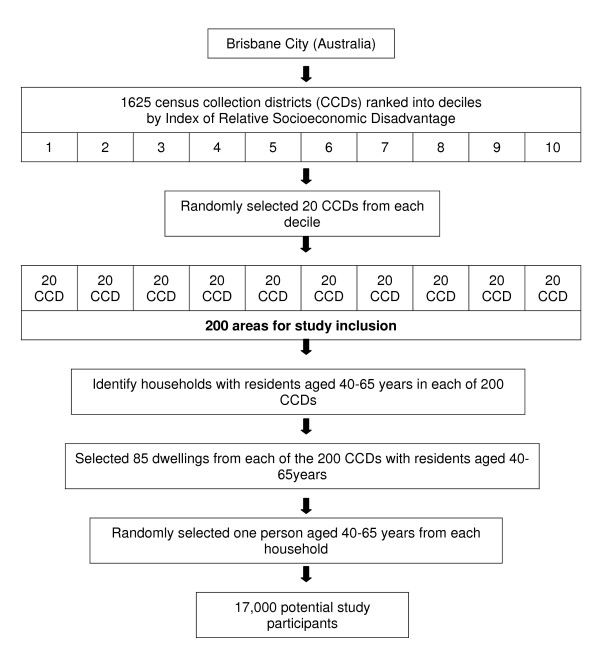
**Overview of sampling procedure to identify HABITAT study areas and participants**.

HABITAT areas and participants were selected using a stratified two stage design. An overview of the process is presented in Figure 1.

#### Study areas

The primary area-level sampling unit was the Census Collection District (CCD). CCDs are the smallest administrative units used by the Australian Bureau of Statistics (ABS) for the collection of census data. In 2001, Brisbane comprised 1680 contiguous CCDs; each CCD contained an average of 203 (SD 81) occupied private dwellings (range 0–697). The ABS assigns those CCDs with a sufficiently large resident population (n = 1655) a socioeconomic score using the Index of Relative Socioeconomic Disadvantage (IRSD). IRSD scores reflect attributes such as the proportion of low income families and individuals with limited educational attainment, and the extent of the workforce in relatively unskilled occupations [23]. To meet the within-CCD sampling targets (see below) we excluded 30 areas that contained very small populations (<50 dwellings) to yield an area-level sampling framework of 1625 CCDs. Based on 2001 census data, the 30 excluded CCDs had a higher proportion of early school leavers (51% and 43%), persons employed in semi- and unskilled occupations (17% and 13%) and low income households (24% and 20%). The 1625 CCDs were ranked by their IRSD scores, divided into deciles, and 20 CCDs were randomly selected from each decile, yielding 200 areas for study inclusion. The sampled (n = 200) and non-sampled CCDs (n = 1425) had similar proportions of early school leavers (44% and 43% respectively), persons employed in semi- and unskilled occupations (14% and 13%) and low income households (19% and 20%).

#### Study participants

Data from the Australian Electoral Commission (AEC) were used to identify all households in each of the selected 200 CCDs that had at least one person aged 40–65 years as at March 2007. In Australia, voting is compulsory for persons aged 18 years and over, so AEC data provides near-complete coverage of the resident population. An average of 85 households per CCD was sampled using systematic without replacement probability proportional-to-size sampling, with size being defined as the number of households in each CCD with at least one person aged 40–65 years. The final stage of the sampling involved randomly selecting one person (of those aged 40–65 years) from each of the 17,000 households (85 × 200).

### Sample Size and Power Calculations

Sample size calculations were conducted via a secondary data analysis of the Victorian Lifestyle and Neighbourhood Environment Study (VicLANES) [24]. VicLANES is a cross-sectional multilevel study of neighborhood socioeconomic status (SES), physical activity, diet, and alcohol consumption involving 4,913 residents of 50 CCDs in Melbourne Australia. Log-transformed minutes of total physical activity varied significantly between (p = 0.019) and within the CCDs (p ≤ 0.000)) after adjustment for age (40–65 years), sex, and education, to give an intra-class correlation (ICC) of 6.3%. Minutes of total physical activity differed significantly between CCDs of low and high SES (p ≤ 0.000). Based on these parameters, *Optimal Design *[25] software was used to compute the power of a two-level multilevel regression analysis to estimate small effect-sizes for the mean difference in physical activity between CCDs of low/high SES. Power was computed for an optimal combination of sample sizes at both the CCD- and individual-level with the ICC constrained to 6.0% and alpha = 0.05. With a sample combination of 200 CCDs and 42 people per CCD the power of the multilevel analysis was 80%, which was sufficient to detect a very small effect size in mean difference in total physical activity among the CCDs. Based on this power analysis, plus estimates of sample retention and loss-to-follow-up from the Australian Longitudinal Study of Women's Health [26] we needed to recruit ~11,200 people in 2007 (56/CCD), ~10,000 in 2009 (50/CCD), and ~8400 in 2011 (42/CCD).

### Data Collection and Study Materials

#### Individual level data

Individual-level data are collected using a structured self-administered mail questionnaire that is ten double sided pages in booklet form (see Additional file 1). Items are grouped into sections to assess

1. Perceptions of the local area (overall rating, ambient surroundings, streets and footpaths, traffic, crime and safety).

2. Proximity of facilities and services from residence (driving time to recreational facilities, walking time to businesses and services).

3. Residential history (length of time at current address, location of previous residence, motivations in moving to local area, place of residence at age 10 and age 25 years).

4. Physical activity (walking, vigorous gardening or housework, vigorous activity, moderate activity, types of recreational activities, use of recreational facilities, occupational activity, cycling for transport, walking for transport).

5. Sedentary behaviour (sitting time in leisure, occupational sitting).

6. Attitudes to activity (intentions, beliefs, efficacy, motivations, barriers).

7. Social influences (social cohesion, social support, professional advice).

8. Health (global rating, physical restrictions, chronic conditions, cigarette smoking, height, weight).

9. Sociodemographic variables (gender, pregnancy, country of birth, educational qualifications, living arrangements, children living in care, employment status, hours worked).

10. Socioeconomic position (occupation, occupation at 25 years, father's occupation, mother's occupation, household income)

11. Other (motor vehicle ownership, dog ownership, use of public transportation).

Items pertaining to neighbourhood perceptions and walking distance to services were adapted from the ANEWS Questionnaire . Items assessing attitudes to activity and social support for activity were adapted from our previous research [27]. Physical activity items were used from the Active Australia survey [2]. Recreational activities were identified from the Exercise, Recreation, and Sport Survey (ERASS). Sedentary behaviour items were adapted from those used in the Australian Longitudinal Study on Women's Health. The social cohesion scale was adapted from Buchner and colleagues [28].

The majority of items use a five or six point likert scale response format, with response options ranging from *strongly disagree *to *strongly agree*. Items assessing time taken to walk or drive to identified destinations use response options of *1–5 minutes, 6–10 minutes, 11–20 minutes, 21–30 minutes, more than 30 minutes*, and *don't know*. Participation in recreational activities (in the previous 12 months) is indicated as *never, rarely, once a month, every two weeks, once a week*, and *more than once a week*. Items assessing motivation to live in the current local area use response options ranging from *not at all important *to *very important*. Social support items use response options ranging from *never *to *very often *and efficacy items use response options ranging from *I know I could not *to *I know I could*. Some items (length of residence, previous residence, time in activity/sitting, cigarette smoking, height and weight, country of birth, occupation, parent's occupation) require participants to write their answer. Other items (health conditions, professional advice, pregnancy, smoking status, dog ownership, motor vehicle ownership) require a yes/no response.

#### Area level data

Area level data were collated using a MapInfo geographic information systems (GIS) database. Data were collected from the local council, National Resources and Water, Energex (electricity supplier), Queensland Transport, the Bureau of Meterology, online databases (such as the telephone book) and environmental audits. Data have been compiled on public transport networks, footpaths, topography, traffic volume, street lights, tree coverage, parks, public services, recreational facilities, fast food chains, and local services (general practice, post office, schools).

### Procedure

Questionnaires are administered using a mail survey method developed by Dillman [29]. For the baseline survey (2007), newspaper advertisements about the study were taken out in the month before the questionnaires were sent. Participants receive a personalized letter advising that the questionnaire will be sent to them in the following week, and highlighting the importance of their response. The questionnaires are mailed in May (2007, 2009, and 2011), with a personalized cover letter, and a pre-addressed pre-paid reply envelope for return. One week later a postcard is mailed to the entire sample, to thank those individuals who return their survey, and remind those who have not to do so. Seven weeks after the initial mail-out, a personalized reminder letter and replacement questionnaire are sent to all non-respondents.

Where possible, GIS data are collected so that it is current at the time of the mail survey data collection. Geographic layers are therefore collected in mid 2007, mid 2009 and mid 2011 so that objective environmental data will complement the mail survey collection periods.

### Cohort Retention and Participant Tracking

Specific strategies are used to engage participants and to maintain contact with the study cohort.

1. Advertisements were placed in local newspapers before the baseline questionnaire was mailed; this was done to describe the study purpose and encourage response.

2. The questionnaires are sent with a personalized cover letter, indicating the importance of response.

3. Follow-up questionnaires (2009, 2011) include a letter with a brief summary of results to show participants how the data are being used and re-emphasize the importance of their continued response.

4. The front page of the questionnaire is titled with the name of the local area and the age of the cohort e.g., *"Living in the Tarragindi Area: A survey about life and recreation for people aged 40–65 years"*.

5. Participants receive a small gratuity (instant scratch-it) with each questionnaire.

6. The questionnaire includes a request for participants to provide the contact details for someone "who will always know where you are if you move".

7. Respondents receive Christmas cards each year, thanking them for their participation. This mailing also includes a change of address card.

8. Participants are sent a newsletter at least every non survey year, which includes summary information about local areas and project results. This mailing also includes a change of address card.

9. Participants can access a project website  to see maps of local areas, and to advise change of address.

10. The electoral roll and Australia Post mail redirection service are searched for the contact details of participants whose questionnaires are received as returned mail. The National Death index is also checked to identify if non-respondents are deceased.

### Measures

#### Primary outcome measures

Primary outcome measures of physical activity include time (minutes/week) spent in walking, walking + moderate activity, vigorous activity, walking for transport, and cycling for transport, as well as an overall measure of activity expenditure (MET.minutes/week). Physical activity participation is assessed using items from the Active Australia surveys that assess the frequency of and total time spent during the last week (i) walking continuously for at least 10 minutes for recreation, exercise, or to get to and from places, (ii) doing vigorous physical activity "which made you breathe harder or puff or pant" e.g., jogging, cycling, aerobics, and (iii) doing moderate physical activity e.g., gentle swimming, social tennis, golf [2]. These items are used for the national monitoring of activity [2], and have acceptable levels of reliability and validity [30,31]. Two additional questions assess time spent walking for transport, and cycling for transport in the previous week.

To minimize measurement error resulting from over-reporting, the time data are truncated at a maximum of 14 hours/week for each domain of activity [2]. Five categories each are derived for walking, and for walking + moderate activity: *none *(<30 mins), *very low *(≥ 30 <90 mins), *low *(≥ 90 <150 mins), *moderate *(≥150 <300 mins) and *high *(>300 mins). Four categories are derived for vigorous activity: *low *(<60 mins), *moderate *(≥ 60 <120 mins), *high *(≥120 <240 mins), and *very high *(>240 mins). For each of these, the moderate category is consistent with activity guidelines which recommend at least 30 minutes of moderate activity on five days of the week, or at least 20 minutes of vigorous activity on three days [1]. Walking for transport is categorized into three levels: *none *(≥ 1 <60 mins), *moderate *(≥ 60 <150 mins), and *high *(≥ 150) and cycling for transport is categorized as *none *(0) and *any *(>0 mins).

An overall measure of energy expenditure is derived by multiplying the time (minutes/week) spent in walking, moderate activity and vigorous activity by an intensity value, and summing the products. Total MET.minutes/week are calculated as [walking minutes * 3METS] + [moderate minutes * 3METS] + [vigorous minutes * 7.5METS]). One MET represents an individual's energy expenditure while sitting quietly. The summed value is then categorized into six levels: *none *(<90), *very low *(≥ 90 <270), *low *(≥ 270 <450), *moderat*e (≥ 450 <900), *high *(≥ 900 <1800) and *very high *(≥ 1800). Again, the moderate category is comparable with activity guidelines to achieve 450–750 MET.mins/week [1].

#### Secondary outcome measures

Physical activity and sedentary behavior are measured using items to assess (i) types of recreational physical activity, (ii) domestic activity, (iii) occupational activity, and (iv) sitting time. Participants are asked to indicate the frequency of doing each of 15 types of recreational physical activity (e.g., exercise class, tennis, swimming) in the last 12 months (*never, once every six months, once a month, once every two weeks, once a week, more than once a week*). Domestic activity is measured using the item from the Active Australia survey that asks about the number of times and total time spent doing vigorous gardening and household activity in the previous week. Occupational activity is measured by asking participants how often they stand, walk and do heavy labour while at work in their main job, with response options of *none of the time, a little of the time, some of the time, most of the time*, and *all of the time*. Sitting time items ask participants to indicate how much time (hours and minutes) they spend sitting, on a usual week day and on a usual weekend day in each of the following situations: traveling to and from places, watching television (including DVDs and games), using the computer at home, and at leisure (e.g., hobbies, reading). A separate item asks employed respondents how much time is spent sitting while at work on a usual day.

#### Determinants measures

**Figure 2:**
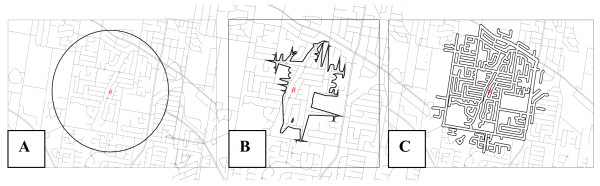
**Examples of catchment areas to be derived to examine area-level characteristics of HABITAT areas**. (a) Circular Buffer (Euclidean) (b) Network Buffer (c) Street Buffer.

Individual-level psychological, social, and environmental factors are measured using scale-scores derived by summing across the relevant questionnaire items (reversing negatively worded items). Area-level measures are based on counts and distance, using Euclidean (or circular), network buffers, and street buffers. Count measures involve the number of variables of interest (e.g., street intersections, recreational facilities, street lights) in relation to each participant's residence. Distance measures involve the euclidean distance in kilometers to the closest variable of interest (e.g., shop, bus stop, train station, recreational facility) from each resident's home. Euclidean or circular buffers query all information contained within a specified radius. For network buffers, the GIS software calculates a specific network distance, in all possible directions, from an origin point (e.g., participants' homes) and creates a buffer connecting the endpoints. A street buffer can be derived from either an euclidean or network buffer and surrounds the streets in the catchment area. The size and nature of the catchments utilized will depend upon the research question under examination. Diagrammatic representations of these different types of catchments are provided in Figure 2. An overview of the types of area-level measures able to be derived is provided in Table 1.

**Table 1:** Examples of HABITAT area-level measures.

**Factor**	**Definition**
Index of Relative Socioeconomic Disadvantage (IRSD)	Reflects socioeconomic attributes such as the proportion of low income families and individuals with limited educational attainment, the occupancy of public sector housing, the unemployment rate, and the extent of the workforce in relatively unskilled occupations, etc.
Street lights	A count of the number of street lights within a defined catchment area of each sampled resident's home
Major street intersections	A count of the number of four- or five-way intersections within a defined catchment around each sampled resident's home. A higher number of intersections indicate greater street connectivity
Hilliness	The standard deviation of the elevation values within a defined catchment area around each sampled resident's home. Larger standard deviation values indicate hillier areas.
Tree coverage	A count of the number of pixels of tree coverage (extracted from aerial photography) multiplied by 2.4, which corresponds to the land-area covered by each pixel. The values for the measure reflect the number of square meters of tree coverage within a defined catchment area around each sampled resident's home
Green space	Number of square meters of public-accessible open green space (e.g. parks, reserves, sports fields) within a defined catchment area around each sampled resident's home
Distance-to-shops	Euclidean distance in kilometers to the closest shop from each sampled residents' home (includes chemist)
Distance-to-public park	Euclidean distance in kilometers to the closest public park from each sampled resident's home
Distance-to-public transport	Euclidean distance in kilometers to the closest bus-stop/train station from each sampled resident's home
Distance to river or coast	Euclidean distance in kilometers to either the Brisbane River or Bay, whichever is closer, from each sampled resident's home
Distance to recreational facility	Euclidean distance in kilometers to a specific recreational facility (e.g., swimming pool, tennis court) from each sampled resident's home
Distance to post box/office	Euclidean distance in kilometers to a post office or postbox from each sampled resident's home
Distance to take away food outlet	Euclidean distance in kilometers to a take away food outlet (includes MacDonalds, Subway, KFC, Hungry Jacks) from each sampled resident's home
Distance to doctor's surgery	Euclidean distance in kilometers to a general practice from each sampled resident's home
Footpaths	Meters of footpath within a defined radius around participant
Pedestrian crossings	Number of pedestrian crossings within a defined catchment area around each sampled residents home
Bike paths	Meters of bike path within a defined catchment area around each sampled resident's home

#### Health variables

Measures are derived from questionnaire items assessing global health status, diagnosed chronic health conditions, smoking status, body mass index (derived from self reported height and weight), and physical restrictions.

#### Sociodemographic measures

Individual-level sociodemographic measures include gender, age, educational qualifications, employment status, occupation group, household composition, gross annual household income, and country of birth. Area level measures will include the index of relative socioeconomic disadvantage.

#### Lifecourse measures

Respondents are asked to provide details about their place of usual residence and their mother's and father's occupation when the respondent was aged 10 and 25 years, and the respondent's main occupation when they were aged 25 years.

### Analyses

Analyses will reflect the design of the sampling and data collection procedure, as well as the research questions considered. The six variables used to measure physical activity are each defined by ordered categories as described above, and this determines the form of the non-linear regression model used to examine associations among variables. For variables with more than two categories, multinomial logit regression models will be used to analyze neighborhood variation in these measures of physical activity as preliminary analyses showed that the more stringent assumptions associated with an ordered cumulative logit model were not met. A binary logit model is appropriate for analyzing the variable *cycling for transport*.

The study design is both multilevel and longitudinal where households are clustered within neighbourhood and data are collected on the same individuals over three time periods. Therefore, multilevel multinomial logit regression models will be used to examine the cross-sectional associations between physical activity and the various sociodemographic, psychological, social, environmental and areal level factors, and these models will be extended to also account for the correlation in observations arising from the longitudinal nature of the design. For example, a random intercept for neighborhoods could be included in the model to account for clustering within neighborhoods and a random intercept for individuals could be included to account for repeated observations over time.

To specifically investigate change in physical activity over time and identify the factors that influence this change, generalized linear latent growth models (GLLGMs) will be used. These models enable the examination of individual trajectories and trajectories of related processes as they change over time. Initially, multilevel regression models and GLLGMs will be estimated separately for each of the measures of physical activity, however, these models will be developed further to include an additional level in the multilevel structure that also accounts for any covariance among the six measures.

## Discussion

Despite recognized physical and psychological health benefits, many mid-aged adults are insufficiently active. Inactivity and low rates of physical activity in mid-age are important public health issues because of the implications for weight status and poor health. In 2001, the prevalence of overweight and obesity in Australia was higher among 45–64 year olds (60% overweight and 21% obese) than any other age group, with almost 7 in 10 men (67%) and more than 5 in 10 women (53%) overweight or obese [32]. As this cohort ages, inactivity and high rates of overweight/obesity will increase the risk of chronic health problems (e.g., cardiovascular disease, diabetes, some cancers, arthritis, depression), and place a significant burden on the health care system. Inactivity has been associated with 7% of disease burden [33] and 18% of all-cause mortality in Australia [34], and 5–10% of deaths and 3–5% of Disability Adjusted Life Years in developed countries [35]. Direct costs of inactivity and obesity account for approximately $5 billion and $4 billion respectively in Canada [36], and 9% of the national health care expenditure in the United States [37].

Understanding the factors enabling and limiting physical activity for this age cohort is therefore, a priority to identify potential mechanisms of change for promotional strategies. Previous research on physical activity influences is limited by cross sectional study designs, and by examining single influences and single domains of physical activity. This paper outlines an innovative study (HABITAT) that redresses these limitations. HABITAT is significant as it

• Uses a longitudinal study design to assess activity adoption, maintenance and discontinuation, as well as the factors associated with activity change.

• Uses a multi-level study design which allows for determination of area level effects over and above individual level effects.

• Focuses on mid-aged adults; this cohort is a priority because of high levels of inactivity and overweight/obesity and the subsequent increased risk of associated chronic disease.

• Includes both mid-aged men and women, unlike other studies that have focused on one gender. This will also allow us to explore gender differences in patterns of activity and activity influences.

• Simultaneously examines psychological, social, environmental, area-level and sociodemographic determinants, rather than focusing on either one determinant domain or one level of measurement. This will facilitate examination of the relative contribution of influences.

• Includes individual-level determinants (e.g., perceptions) and area-level determinants (e.g., neighborhood features) which will allow for comparison of subjective and objective data such as the availability of facilities and neighborhood features.

• Measures physical activity across several domains, with primary outcome measures including walking in general, moderate activity, vigorous activity, walking for transport, and cycling for transport. Secondary outcome measures include participation in specific types of recreational activities, domestic activity, occupational activity, and sitting time. This will facilitate the development of more behaviorally specific predictive models.

• Assesses the impact of individual-level socioeconomic position and area-level disadvantage, which are potentially important covariates of physical activity and potential effect modifiers of the associations between determinants and physical activity.

### Limitations

When interpreting HABITAT findings, consideration needs to be given to a number of methodological issues. The physical activity data are based on self-report, which is prone to bias and measurement error that can result in an under estimation in the level of physical activity both among sedentary populations and for walking [38]. Self-report physical activity data are however, pragmatic for large samples and considered appropriate for population monitoring [38], and are reliably associated with morbidity and mortality outcomes [39]. Assessment of the reliability and validity of survey items with objective data from pedometers and accelerometers has indicated acceptable levels for population based studies [30].

The generalizability of results may be limited by errors associated with the sampling frame for study participants, mail survey data collection, survey non-response, and item non response. HABITAT participants were identified from the electoral roll, which can under-represent transient, migrant, and socially disadvantaged individuals [40]. Mail survey methodologies may yield less complete data than interview studies [41], with both survey and item non-response more prevalent among individuals of low socioeconomic position [42,43]. The sociodemographic profile of participants will be compared with census data to assess representativeness.

GIS data relies on either aerial photography or on individuals who periodically go out into the field and create or update digital maps. Many of the GIS layers of data used from secondary sources are presumed to be current when they are supplied to HABITAT, i.e., if we receive the information in 2007 we assume it is current for that year. However, some layers, such as tree coverage from aerial photography, are from data collected earlier than when it is supplied to HABITAT. There may be therefore, a time gap between GIS data and mail survey data. This may have implications when (for example) comparing subjective perceptions of the environment with actual measures. Some of the environmental data have been validated by audits, which will be reported elsewhere.

## Conclusion

Understanding the interplay of psychological, social, environmental, area-level and sociodemographic influences on physical activity can guide health promotion [44]. By understanding the relative importance of the different influences on physical activity, priorities can be identified in order to effectively direct and shape strategies to focus on specific and salient correlates that account for maximum amounts of variation in physical activity participation. The information provided by HABITAT is important as it will identify priority "place" and "people" targets for public policy, health policy, and health promotion aimed at increasing physical activity among mid-aged men and women, thereby maximizing effectiveness of interventions and optimizing the use of limited resources. This knowledge is crucial for the development of strategies to increase physical activity in mid-aged adults, and minimize the myriad of inactivity-related health problems that can develop in mid to late adulthood.

## Abbreviations

AEC: Australian Electoral Commission; CCD: Census Collection District; GIS: Geographic Information Systems; ICC: Intra-class correlation; IRSD: Index of Relative Socioeconomic Disadvantage; NHMRC: (Australian) National Health and Medical Research Committee; SES: Socioeconomic status.

## Competing interests

The authors declare that they have no competing interests.

## Authors' contributions

NB led the writing for this manuscript, and wrote the introduction, discussion, and measures. GT and MH wrote the methods and analyses section. LW wrote sections relating to the GIS area-level data. All authors contributed to the design, sampling and data collection protocol, and wrote sections of the grant applications that were used in the paper. All authors read and approved the manuscript.

## Pre-publication history

The pre-publication history for this paper can be accessed here:



## Supplementary Material

Additional file 1 **HABITAT 2007 Questionnaire**. A survey about life and recreation for people aged 40–65 years Click here for file

## References

[B1] Haskell WL, Lee I-M, Pate R, Powell K, Blair S, Franklin B, Macera C, Heath G, Thompson P, Bauman A (2007). Physical Activity and Public Health: Updated Recommendation for Adults from the American College of Sports Medicine and the American Heart Association. Medicine and Science in Sports and Exercise.

[B2] Armstrong T, Bauman A, Davies J (2000). Physical activity patterns of Australian adults. Results of the 1999 National Physical Activity Survey.

[B3] Guthold RO, Strong T, Chatterji KL, Morabia SA (2008). Worldwide variability in physical inactivity – A 51-country survey. American Journal of Preventive Medicine.

[B4] Stamatakis E, Ekelund U, Wareham N (2007). Temporal trends in physical activity in England: The Health Survey for England 1991 to 2004. Preventive Medicine.

[B5] Stokols D (1996). Translating social ecological theory into guidelines for community health promotion. American Journal of Health Promotion.

[B6] Breslow L (1996). Social ecological strategies for promoting healthy lifestyles. American Journal of Health Promotion.

[B7] Trost SG, Owen N, Bauman AE, Sallis JF, Brown WJ (2002). Correlates of adult's participation in physical activity: review and update. Medicine & Science in Sports & Exercise.

[B8] Seefeldt V, Malina RM, Clark MA (2002). Factors affecting levels of physical activity in adults. Sports Medicine.

[B9] Bauman AE, Sallis JF, Dzewaltowski DA, Owen N (2002). Toward a better understanding of the influences on physical activity: The role of determinants, correlates, causal variables, mediators, moderators, and confounders. American Journal of Preventive Medicine.

[B10] Sherwood NE, Jeffery RW (2000). The behavioral determinants of exercise: Implications for physical activity interventions. Annual Review of Nutrition.

[B11] Buckworth J (2000). Exercise determinants and interventions. International Journal of Sport Psychology.

[B12] Brown WJ, Burton NW, Heesch KC (2008). Physical activity and health in mid age and older Australian women.

[B13] Bouchard C, Shephard RJ, Stephens T, Bouchard C, Shephard RJ, Stephens T (1993). Physical activity, fitness, and health: status and determinants. Physical activity, fitness and health Consensus statement.

[B14] Dishman RK, Singer RN, Murphy M, Tennant LK (1993). Exercise adherence. Handbook of research on sport psychology.

[B15] Dishman RK, (Ed) (1994). Advances in exercise adherence.

[B16] Baranowski T, Anderson C, Carmack C (1998). Mediating variable framework in physical activity interventions. How are we doing? How might we do better?. American Journal of Preventive Medicine.

[B17] Laitakari J, Vuori I, Oja P (1996). Is long-term maintenance of health-related physical activity possible? An analysis of concepts and evidence. Health Education Research.

[B18] De Bourdeaudhuij I, Sallis J (2002). Relative contribution of psychosocial variables to the explanation of physical activity in three population based adult samples. Preventive Medicine.

[B19] Giles-Corti B, Donovan RJ (2002). The relative influence of individual, social, and physical environment determinants of physical activity. Social Science & Medicine.

[B20] Sallis J, Haskell WL, Fortmann SP, Vranizan KM, Taylor CB, Solomon DS (1986). Predictors of adoption and maintenance of physical activity in a community sample. Preventive Medicine.

[B21] Humpel N, Owen N, Iverson D, Leslie E, Bauman A (2004). Perceived environmental attributes, residential location and walking for particular purposes. American Journal of Preventive Medicine.

[B22] Burton NW, Turrell G, Oldenburg B, Sallis J (2005). The relative contributions of psychological, social, and environmental variables to explain participation in walking, moderate-, and vigorous-intensity leisure-time physical activity. Journal of Physical Activity and Health.

[B23] Australian Bureau of Statistics (2003). Socio-economic Indexes for Areas, Australia, 2001.

[B24] Kavanagh AM, Goller JL, King T, Jolley D, Crawford D, Turrell G (2005). Urban area disadvantage and physical activity: a multilevel study in Melbourne, Australia. Journal of Epidemiology & Community Health.

[B25] HLM Software (2006). Optimal Design for Multilevel and Longitudinal Research.

[B26] Lee C, Dobson AJ, Brown WJ, Bryson L, Byles J, Warner-Smith P, Young AF (2005). Cohort Profile: the Australian Longitudinal Study on Women's Health. International Journal of Epidemiology.

[B27] Burton NW, Oldenburg B, Sallis JF, Turrell G (2007). Measuring psychological, social, and environmental influences on leisure-time physical activity. Australian and New Zealand Journal of Public Health.

[B28] Buckner J (1988). The development of an instrument to measure neighborhood cohesion. American Journal of Community Psychology.

[B29] Dillman DA (2000). Mail and internet surveys: the tailored design method.

[B30] Brown WB, Burton NW, Marshall AL, Miller YD (2008). Reliability and validity of a modified self-administered version of the Active Australia physical 2 activity survey in a sample of mid-age women. Australian and New Zealand Journal of Public Health.

[B31] Brown WJ, Bauman A, Chey T, Trost SG, Mummery WK (2004). Comparison of surveys used to measure physical activity. Australian and New Zealand Journal of Public Health.

[B32] O'Brien K, Webbie K, Australian Institute of Health and Welfare (2003). Are all Australians gaining weight? Differentials in overweight and obesity among adults, 1989–90 to 2001. Bulletin No. 11. AIHW Cat. No. AUS 39.

[B33] Mathers CD, Vos ET, Stevenson CE, Begg SJ (2000). The Australian burden of disease study: measuring the loss of health from diseases, injuries and risk factors. Medical Journal of Australia.

[B34] Stephenson J, Bauman A, Armstrong T, Smith B, Bellew B (2000). The costs of illness attributable to physical inactivity in Australia: a preliminary study.

[B35] World Health Organisation (2002). The World Health Report, 2002. Reducing risk and promoting healthy life.

[B36] Katzmarzyk PT, Janssen I (2004). The economic costs associated with physical inactivity and obesity in Canada: an update. Canadian Journal of Applied Physiology.

[B37] Colditz GA (1999). Economic costs of obesity and inactivity. Medicine & Science in Sports & Exercise.

[B38] Tudor-Locke CE, Myers AM (2001). Challenges and opportunities for measuring physical activity in sedentary adults. Social Science & Medicine.

[B39] Bauman AE (2004). Updating the evidence that physical activity is good for health: an epidemiological review 2000–2003. Journal of Science and Medicine in Sport.

[B40] Turrell G, Najman JM (1995). Collecting food-related data from low socioeconomic groups: how adequate are our current research designs?. Australian Journal of Public Health.

[B41] Van Campen C, Sixma M, Kerssens JJ, Peters L (1998). Comparisons of the costs and quality of patient data collection by mail versus telephone versus in-person interviews. European Journal of Public Health.

[B42] Burton NW, Turrell G, Oldenburg B (2004). Item non-response in a population-based study of physical activity. Physical Activity and Health.

[B43] Van Loon AJM, Tijhuis M, Picavet HSJ, Surtees PG, Ormel J (2003). Survey non-response in the Netherlands: effects on prevalence estimates and associations. Annals of Epidemiology.

[B44] McGinnis JM, Williams-Russo G, Knickman JR (2002). The case for more active policy attention to health promotion. Health Affairs.

