# Supplementary material for: HABITAT: A longitudinal multilevel study of physical activity change in mid-aged adults
Source: BMC Public Health. 2009 Mar 5;9:76. doi: 10.1186/1471-2458-9-76 (PMC2664805; doi:10.1186/1471-2458-9-76)
Supplement: Additional file 1 — HABITAT 2007 Questionnaire. A survey about life and recreation for people aged 40–65 years [file 1471-2458-9-76-S1.pdf]

HABITAT

how areas in brisbane influence health and activity

Living in the Seventeen Mile Rocks Area

A SURVEY ABOUT LIFE AND RECREATION FOR PEOPLE AGED 40-65 YEARS

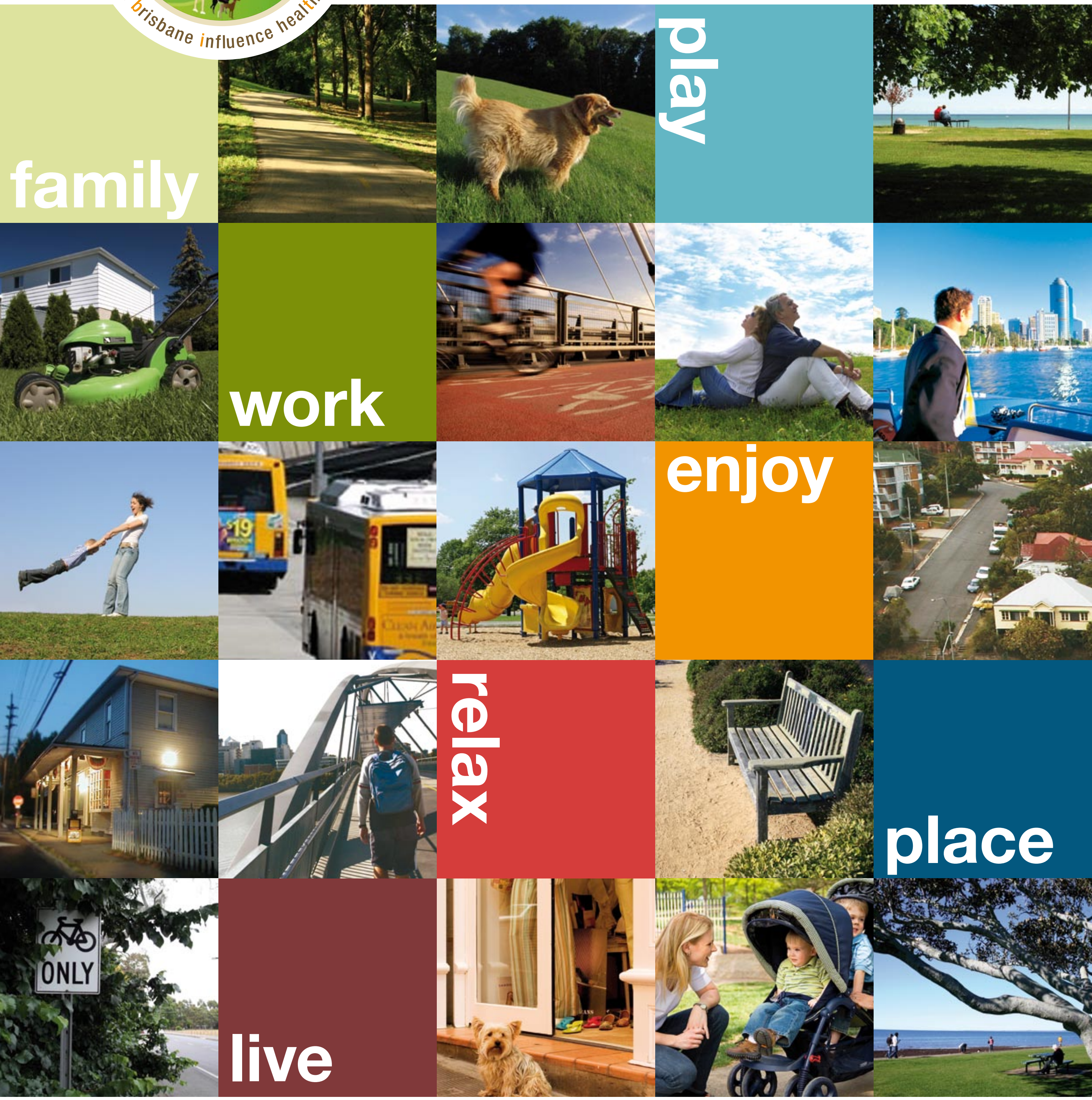

Queensland University of Technology

The University Of Queensland

This project is funded by the National Health and Medical Research Council and supported by the National Heart Foundation

HABITAT

how areas in brisbane influence health and activity

We greatly appreciate your help with this survey.
Your answers are very important to us.

Please remember:

- There are no right or wrong answers: we just want to know what YOU think
- Provide only one answer for each item and please don't skip any questions
- Your answers will be treated as strictly **PRIVATE AND CONFIDENTIAL**

If you have any questions:
Please call our Freecall number on 1800 452 543

Once you have completed the survey, please return it in the enclosed reply paid envelope (no stamps necessary).

Many questions in this survey ask about your suburb. When we talk about 'your suburb' we ask you to think about living in the Seventeen Mile Rocks area.

Section 1:

YOU AND YOUR SUBURB

1. Overall, how would you rate your suburb as a place to live?

Please tick one.	Excellent	Very good	Good	Fair	Poor
	☐ 1	☐ 2	☐ 3	☐ 4	☐ 5

2. The following statements are about your **suburb and the people living around you**. How much do you agree or disagree with each statement?

Please tick the box that best applies to you and your suburb.	Strongly disagree	Disagree	Unsure	Agree	Strongly agree
a) I have a lot in common with many people in my suburb	☐ 1	☐ 2	☐ 3	☐ 4	☐ 5
b) If I no longer lived here, hardly anyone around here would notice	☐ 1	☐ 2	☐ 3	☐ 4	☐ 5
c) I am good friends with many people in my suburb	☐ 1	☐ 2	☐ 3	☐ 4	☐ 5
d) I generally trust my neighbours to look out for my property	☐ 1	☐ 2	☐ 3	☐ 4	☐ 5
e) I have little to do with most people in my suburb	☐ 1	☐ 2	☐ 3	☐ 4	☐ 5

3. The following statements are about **traffic** in your suburb. How much do you agree or disagree with each statement?

Please tick the box that best applies to your suburb.	Strongly disagree	Disagree	Unsure	Agree	Strongly agree
a) In my suburb, there is usually a lot of traffic on the local streets	☐ 1	☐ 2	☐ 3	☐ 4	☐ 5
b) The speed of traffic on most nearby streets is usually slow (50kph or less)	☐ 1	☐ 2	☐ 3	☐ 4	☐ 5
c) There are many traffic slowing devices in my suburb such as speed humps, roundabouts, traffic islands	☐ 1	☐ 2	☐ 3	☐ 4	☐ 5
d) I live on or near a main road or busy throughway for motor vehicles	☐ 1	☐ 2	☐ 3	☐ 4	☐ 5
e) In my suburb there are a lot of exhaust fumes from motor vehicles	☐ 1	☐ 2	☐ 3	☐ 4	☐ 5

4. The following statements are about your suburb's **surroundings**. How much do you agree or disagree with each statement?

Please tick the box that best applies to your suburb.	Strongly disagree	Disagree	Unsure	Agree	Strongly agree
a) There is lots of greenery around my suburb (trees, bushes, household gardens)	☐ 1	☐ 2	☐ 3	☐ 4	☐ 5
b) There are many interesting things to look at in my suburb	☐ 1	☐ 2	☐ 3	☐ 4	☐ 5
c) There is tree cover along many of the footpaths in my suburb	☐ 1	☐ 2	☐ 3	☐ 4	☐ 5
d) My suburb is generally free from litter or rubbish	☐ 1	☐ 2	☐ 3	☐ 4	☐ 5
e) There are attractive buildings and homes in my suburb	☐ 1	☐ 2	☐ 3	☐ 4	☐ 5
f) There are pleasant natural features in my suburb (e.g. nature reserves, beach, riverfront, bushland)	☐ 1	☐ 2	☐ 3	☐ 4	☐ 5
g) My suburb is generally free from graffiti	☐ 1	☐ 2	☐ 3	☐ 4	☐ 5

5. The following statements are about **streets and footpaths** in your suburb. How much do you agree or disagree with each statement?

Please tick the box that best applies to your suburb.	Strongly disagree	Disagree	Unsure	Agree	Strongly agree
a) Many streets in my suburb have cul-de-sacs (dead-end streets)	☐ 1	☐ 2	☐ 3	☐ 4	☐ 5
b) There are footpaths on most of the streets in my suburb	☐ 1	☐ 2	☐ 3	☐ 4	☐ 5
c) There are many four-way intersections in my suburb	☐ 1	☐ 2	☐ 3	☐ 4	☐ 5
d) Many streets in my suburb are hilly	☐ 1	☐ 2	☐ 3	☐ 4	☐ 5
e) Most footpaths in my suburb are well lit at night	☐ 1	☐ 2	☐ 3	☐ 4	☐ 5
f) Many roads and streets in my suburb have pedestrian crossings and traffic signals	☐ 1	☐ 2	☐ 3	☐ 4	☐ 5
g) Most of the footpaths in my suburb are well maintained (flat and even, not broken or cracked)	☐ 1	☐ 2	☐ 3	☐ 4	☐ 5

6. The following statements are about **crime and safety** in your suburb. How much do you agree or disagree with each statement?

Please tick the box that best applies to your suburb.	Strongly disagree	Disagree	Unsure	Agree	Strongly agree
a) There is a lot of crime in my suburb	☐ 1	☐ 2	☐ 3	☐ 4	☐ 5
b) There are unsecured dogs in my suburb	☐ 1	☐ 2	☐ 3	☐ 4	☐ 5
c) Children are safe walking around the suburb during the day	☐ 1	☐ 2	☐ 3	☐ 4	☐ 5
d) The level of crime in my suburb makes it unsafe to walk on the streets at night	☐ 1	☐ 2	☐ 3	☐ 4	☐ 5
e) There are rowdy youth on the streets or hanging around in parks in my suburb	☐ 1	☐ 2	☐ 3	☐ 4	☐ 5
f) The level of crime in my suburb makes it unsafe to walk on the streets during the day	☐ 1	☐ 2	☐ 3	☐ 4	☐ 5
g) In my suburb, I would feel safe walking home from a bus stop or train station at night	☐ 1	☐ 2	☐ 3	☐ 4	☐ 5

Section 2:

FACILITIES AND SERVICES IN YOUR SUBURB

This next section is about recreation facilities, businesses and services that might be in your suburb.

7. About how long would it take to **DRIVE** from your home to the **NEAREST** recreation facility listed below? **(Please think of the closest one.)**

Please tick one box for each item.	1-5 minutes	6-10 minutes	11-20 minutes	21-30 minutes	More than 30 minutes	Don't know
a) Bike path	☐ 1	☐ 2	☐ 3	☐ 4	☐ 5	☐ 6
b) Oval or sports field	☐ 1	☐ 2	☐ 3	☐ 4	☐ 5	☐ 6
c) Public park	☐ 1	☐ 2	☐ 3	☐ 4	☐ 5	☐ 6
d) Golf course	☐ 1	☐ 2	☐ 3	☐ 4	☐ 5	☐ 6
e) Public swimming pool	☐ 1	☐ 2	☐ 3	☐ 4	☐ 5	☐ 6
f) Gym or fitness centre	☐ 1	☐ 2	☐ 3	☐ 4	☐ 5	☐ 6
g) Public tennis court	☐ 1	☐ 2	☐ 3	☐ 4	☐ 5	☐ 6
h) Ocean beach	☐ 1	☐ 2	☐ 3	☐ 4	☐ 5	☐ 6
i) Indoor sports centre	☐ 1	☐ 2	☐ 3	☐ 4	☐ 5	☐ 6
j) Lawn bowls club	☐ 1	☐ 2	☐ 3	☐ 4	☐ 5	☐ 6
k) River	☐ 1	☐ 2	☐ 3	☐ 4	☐ 5	☐ 6
l) Public recreation area (e.g. Boondall Wetlands, Brisbane Forest Park, Mt Cootha Reserve)	☐ 1	☐ 2	☐ 3	☐ 4	☐ 5	☐ 6

8. About how long would it take you to **WALK** from your home to the **NEAREST** business or facility listed below? **(Please think of the closest one.)**

Please tick one box for each item.	1-5 minutes	6-10 minutes	11-20 minutes	21-30 minutes	More than 30 minutes	Don't know
a) Supermarket	☐ 1	☐ 2	☐ 3	☐ 4	☐ 5	☐ 6
b) Fruit and Veg shop	☐ 1	☐ 2	☐ 3	☐ 4	☐ 5	☐ 6
c) Bike path	☐ 1	☐ 2	☐ 3	☐ 4	☐ 5	☐ 6
d) Post Office	☐ 1	☐ 2	☐ 3	☐ 4	☐ 5	☐ 6
e) Library	☐ 1	☐ 2	☐ 3	☐ 4	☐ 5	☐ 6
f) Primary school	☐ 1	☐ 2	☐ 3	☐ 4	☐ 5	☐ 6
g) High school	☐ 1	☐ 2	☐ 3	☐ 4	☐ 5	☐ 6
h) Café/restaurant	☐ 1	☐ 2	☐ 3	☐ 4	☐ 5	☐ 6
i) Chemist	☐ 1	☐ 2	☐ 3	☐ 4	☐ 5	☐ 6
j) Bus stop	☐ 1	☐ 2	☐ 3	☐ 4	☐ 5	☐ 6
k) Train station	☐ 1	☐ 2	☐ 3	☐ 4	☐ 5	☐ 6
l) Public Park	☐ 1	☐ 2	☐ 3	☐ 4	☐ 5	☐ 6
m) Liquor store/bottle shop	☐ 1	☐ 2	☐ 3	☐ 4	☐ 5	☐ 6
n) Doctor/medical centre	☐ 1	☐ 2	☐ 3	☐ 4	☐ 5	☐ 6
o) Ferry terminal	☐ 1	☐ 2	☐ 3	☐ 4	☐ 5	☐ 6
p) Childcare centre	☐ 1	☐ 2	☐ 3	☐ 4	☐ 5	☐ 6
q) Post box	☐ 1	☐ 2	☐ 3	☐ 4	☐ 5	☐ 6
r) Oval or sports field	☐ 1	☐ 2	☐ 3	☐ 4	☐ 5	☐ 6
s) Pub, hotel or tavern	☐ 1	☐ 2	☐ 3	☐ 4	☐ 5	☐ 6

Section 3:

REASONS FOR MOVING TO YOUR SUBURB

9. How long have you lived at your current address?

Years		Months		Weeks
	OR		OR	

10. Where did you live immediately before your current address?

Country	State/Territory	City/Town	Suburb/Postcode

11. How important were each of the following in your decision to move to your **current** suburb?

Please tick one box for each item.	Not at all important	A little important	Somewhat important	Quite important	Very important
a) Affordability of land, housing or rent	1	2	3	4	5
b) Closeness to open space (e.g. parks)	1	2	3	4	5
c) Ease of walking to places	1	2	3	4	5
d) Sense of community	1	2	3	4	5
e) Closeness to schools	1	2	3	4	5
f) Safety from crime	1	2	3	4	5
g) Closeness to public transport	1	2	3	4	5
h) Wanted to live close to shops	1	2	3	4	5
i) Access to freeways or main roads	1	2	3	4	5
j) Closeness to work	1	2	3	4	5
k) Closeness to recreational facilities	1	2	3	4	5
l) Closeness to childcare	1	2	3	4	5
m) Closeness to relatives	1	2	3	4	5
n) Closeness to city	1	2	3	4	5
o) Near to green space/bushland	1	2	3	4	5
p) Moved in with my spouse/partner	1	2	3	4	5
q) Investment potential	1	2	3	4	5
r) Other *(please describe)*					

Section 4:

ACTIVITY AND RECREATION

The next questions are about any physical activities that you may have done in the **LAST WEEK**:

12\. a) In the **LAST WEEK**, how many times have you walked **continuously, for at least 10 minutes**, for recreation, exercise, or to get to or from places?

Write in number ____ *If NONE, please write 0*

b) What do you estimate was the total time that you spent walking in this way in the **LAST WEEK**?

Hours ____ *Minutes* ____

13\. a) In the **LAST WEEK**, how many times did you do any vigorous gardening or heavy work around the yard, which made you breathe harder or puff and pant?

Write in number ____ *If NONE, please write 0*

b) What do you estimate was the total time that you spent doing vigorous gardening or heavy work around the yard in the **LAST WEEK**?

Hours ____ *Minutes* ____

The next questions **EXCLUDE** household chores, gardening, or yard work:

14\. a) In the **LAST WEEK**, how many times did you do any vigorous physical activity which made you breathe harder or puff and pant?

Examples: Jogging, cycling, aerobics, competitive tennis

Write in number ____ *If NONE, please write 0*

b) What do you estimate was the total time that you spent doing this vigorous physical activity in the **LAST WEEK**?

Hours ____ *Minutes* ____

15\. a) In the **LAST WEEK**, how many times did you do any other more moderate physical activities that you have not already mentioned?

Examples: Gentle swimming, social tennis, golf

Write in number ____ *If NONE, please write 0*

b) What do you estimate was the total time that you spent doing these activities in the **LAST WEEK**?

Hours ____ *Minutes* ____

16. Here is a list of recreational activities. How often have you done these in the last **TWELVE** (12) months?

Please tick one box for each activity.	Never	Once every six months	Once a month	Once every two weeks	Once a week	More than once a week
a) Physical activity with others in a park (e.g. frisbee, games)	☐ 1	☐ 2	☐ 3	☐ 4	☐ 5	☐ 6
b) Running or jogging	☐ 1	☐ 2	☐ 3	☐ 4	☐ 5	☐ 6
c) Weights	☐ 1	☐ 2	☐ 3	☐ 4	☐ 5	☐ 6
d) Cycling	☐ 1	☐ 2	☐ 3	☐ 4	☐ 5	☐ 6
e) Exercise class (e.g. aerobics)	☐ 1	☐ 2	☐ 3	☐ 4	☐ 5	☐ 6
f) Golf	☐ 1	☐ 2	☐ 3	☐ 4	☐ 5	☐ 6
g) Swimming	☐ 1	☐ 2	☐ 3	☐ 4	☐ 5	☐ 6
h) Tennis	☐ 1	☐ 2	☐ 3	☐ 4	☐ 5	☐ 6
i) Team sports (e.g. football, netball, hockey, softball)	☐ 1	☐ 2	☐ 3	☐ 4	☐ 5	☐ 6
j) Yoga, Pilates, tai chi or qigong	☐ 1	☐ 2	☐ 3	☐ 4	☐ 5	☐ 6
k) Lawn bowls	☐ 1	☐ 2	☐ 3	☐ 4	☐ 5	☐ 6
l) Home-based exercises (e.g. stretches, fitball, workout video)	☐ 1	☐ 2	☐ 3	☐ 4	☐ 5	☐ 6
m) Boating/sailing	☐ 1	☐ 2	☐ 3	☐ 4	☐ 5	☐ 6
n) Water activities (e.g. rowing, diving, canoeing)	☐ 1	☐ 2	☐ 3	☐ 4	☐ 5	☐ 6
o) Physical activity with others on a beach (e.g. frisbee, games)	☐ 1	☐ 2	☐ 3	☐ 4	☐ 5	☐ 6

17. This question asks about how much time you spend **SITTING** in the following situations **ON A USUAL DAY** (if NONE, please write 0):

	On a WEEK DAY		On a WEEKEND DAY	
a) While travelling to and from places	Hours	Minutes	Hours	Minutes
b) While watching television (including DVDs, videos, Xbox and PlayStation)	Hours	Minutes	Hours	Minutes
c) While using the computer at home	Hours	Minutes	Hours	Minutes
d) In your leisure time, NOT including TV and the computer (e.g. hobbies, reading, dining out)	Hours	Minutes	Hours	Minutes

18. **Have you ever used any of the following recreational facilities?** If no, please tick 'never'. If yes, tick the box showing how recently **AND** give the name of the facility and the suburb it is in.

	No, never	Yes, in the last month	Yes, 1-12 months ago	Yes, more than a year ago	Name of main facility you used	Which suburb is the facility in?
a) Public swimming pool	☐ 1	☐ 2 OR	☐ 3 OR	☐ 4		
b) Indoor recreation facility (e.g. gym, indoor sports, yoga centre)	☐ 1	☐ 2 OR	☐ 3 OR	☐ 4		
c) Oval or sporting field	☐ 1	☐ 2 OR	☐ 3 OR	☐ 4		
d) Outdoor recreation facility (e.g. golf course, tennis court)	☐ 1	☐ 2 OR	☐ 3 OR	☐ 4		
e) Public park	☐ 1	☐ 2 OR	☐ 3 OR	☐ 4		
f) Public recreation area (e.g. Boondall Wetlands, Brisbane Forest Park, Mt Cootha Reserve)	☐ 1	☐ 2 OR	☐ 3 OR	☐ 4		
g) Bike path	☐ 1	☐ 2 OR	☐ 3 OR	☐ 4		

Section 5:

YOUR THOUGHTS AND FEELINGS ABOUT PHYSICAL ACTIVITY

This section asks for your personal opinion about physical activity. This includes things like walking, sports, running, swimming, cycling, etc. There are no right or wrong answers.

19. How strong or weak is your intention to be physically active?

Please tick one.	Very weak	Weak	Unsure	Strong	Very strong
	☐ 1	☐ 2	☐ 3	☐ 4	☐ 5

20. To what extent do you agree or disagree with each statement?

Please tick one box per item.	Strongly disagree	Disagree	Unsure	Agree	Strongly agree
a) Doing some kind of physical activity is a habit for me	☐ 1	☐ 2	☐ 3	☐ 4	☐ 5
b) Physical activity takes a lot of effort	☐ 1	☐ 2	☐ 3	☐ 4	☐ 5
c) Right now, I am better off spending my time doing other things than physical activity	☐ 1	☐ 2	☐ 3	☐ 4	☐ 5
d) In the last 2 years, I have been involved in regular physical activity at one time or another	☐ 1	☐ 2	☐ 3	☐ 4	☐ 5
e) At school I did well at sport	☐ 1	☐ 2	☐ 3	☐ 4	☐ 5
f) Being physically active is important to me	☐ 1	☐ 2	☐ 3	☐ 4	☐ 5
g) Doing physical activity requires serious commitment	☐ 1	☐ 2	☐ 3	☐ 4	☐ 5
h) I am the type of person who likes to have a go at things	☐ 1	☐ 2	☐ 3	☐ 4	☐ 5
i) I have always done some kind of physical activity	☐ 1	☐ 2	☐ 3	☐ 4	☐ 5
j) After a hard day I don't need to do physical activity, I need to relax	☐ 1	☐ 2	☐ 3	☐ 4	☐ 5
k) I have always been good at sport/physical activity	☐ 1	☐ 2	☐ 3	☐ 4	☐ 5
l) I have never been the type to sit still for too long	☐ 1	☐ 2	☐ 3	☐ 4	☐ 5
m) Physical activity is hard work	☐ 1	☐ 2	☐ 3	☐ 4	☐ 5
n) I am not the physically active type	☐ 1	☐ 2	☐ 3	☐ 4	☐ 5
o) I get all the physical activity I need from being busy during the day	☐ 1	☐ 2	☐ 3	☐ 4	☐ 5

21. Do you think that you could do physical activity regularly when:

Please tick one box for each item.

	I know I could not ←		Maybe I could		→ I know I could
a) You have chores to do	☐ 1	☐ 2	☐ 3	☐ 4	☐ 5
b) You are feeling sad or depressed	☐ 1	☐ 2	☐ 3	☐ 4	☐ 5
c) You have had a long, tiring day	☐ 1	☐ 2	☐ 3	☐ 4	☐ 5
d) Your family wants more time with you	☐ 1	☐ 2	☐ 3	☐ 4	☐ 5
e) You have work demands	☐ 1	☐ 2	☐ 3	☐ 4	☐ 5
f) You have social commitments	☐ 1	☐ 2	☐ 3	☐ 4	☐ 5

22. During the past 3 months, how often have family or friends:

Please tick one box for each item.

	Never	Rarely	Some-times	Often	Very often
a) Encouraged you to do physical activity	☐ 1	☐ 2	☐ 3	☐ 4	☐ 5
b) Criticised you or made fun about you doing physical activity	☐ 1	☐ 2	☐ 3	☐ 4	☐ 5
c) Done something to help you be physically active	☐ 1	☐ 2	☐ 3	☐ 4	☐ 5
d) Done or offered to do physical activity with you	☐ 1	☐ 2	☐ 3	☐ 4	☐ 5
e) Made it difficult for you to do physical activity	☐ 1	☐ 2	☐ 3	☐ 4	☐ 5
f) Invited you to do physical activity with them	☐ 1	☐ 2	☐ 3	☐ 4	☐ 5
g) Discussed physical activity with you	☐ 1	☐ 2	☐ 3	☐ 4	☐ 5
h) Complained about you doing physical activity	☐ 1	☐ 2	☐ 3	☐ 4	☐ 5

23. There are different reasons why people might do physical activity. Which of these could motivate **YOU** to do physical activity?

Please tick one box for each item.

	Strongly disagree	Disagree	Unsure	Agree	Strongly agree
a) To prevent health problems	☐ 1	☐ 2	☐ 3	☐ 4	☐ 5
b) To help manage stress	☐ 1	☐ 2	☐ 3	☐ 4	☐ 5
c) To lose weight, or manage my weight	☐ 1	☐ 2	☐ 3	☐ 4	☐ 5
d) To spend time with others (e.g. friends, family, partner)	☐ 1	☐ 2	☐ 3	☐ 4	☐ 5
e) To improve my appearance	☐ 1	☐ 2	☐ 3	☐ 4	☐ 5
f) To make me feel good	☐ 1	☐ 2	☐ 3	☐ 4	☐ 5

24. Here are some things that might make it difficult to do physical activity. Which of these things make it difficult for **YOU** to do physical activity?

Please tick one box for each item.

	Strongly disagree	Disagree	Unsure	Agree	Strongly agree
a) Lack of money	☐ 1	☐ 2	☐ 3	☐ 4	☐ 5
b) Poor health	☐ 1	☐ 2	☐ 3	☐ 4	☐ 5
c) Facilities are too far away	☐ 1	☐ 2	☐ 3	☐ 4	☐ 5
d) Problems with transport	☐ 1	☐ 2	☐ 3	☐ 4	☐ 5
e) My age	☐ 1	☐ 2	☐ 3	☐ 4	☐ 5
f) Cost of membership/equipment	☐ 1	☐ 2	☐ 3	☐ 4	☐ 5
g) I do not enjoy physical activity	☐ 1	☐ 2	☐ 3	☐ 4	☐ 5
h) Lack of time	☐ 1	☐ 2	☐ 3	☐ 4	☐ 5
i) I have a disability	☐ 1	☐ 2	☐ 3	☐ 4	☐ 5
j) I'm too shy or embarrassed	☐ 1	☐ 2	☐ 3	☐ 4	☐ 5
k) My weight	☐ 1	☐ 2	☐ 3	☐ 4	☐ 5
l) Lack of access to childcare	☐ 1	☐ 2	☐ 3	☐ 4	☐ 5
m) Lack of skill	☐ 1	☐ 2	☐ 3	☐ 4	☐ 5
n) Work demands	☐ 1	☐ 2	☐ 3	☐ 4	☐ 5

Section 6:

GENERAL HEALTH AND LIFESTYLE

25. In general, would you say your health is:

Please tick one.	Excellent	Very good	Good	Fair	Poor
	☐ 1	☐ 2	☐ 3	☐ 4	☐ 5

26. In the last year, how often:

Please tick one box for each item.	None of the time	A little of the time	Some of the time	Most of the time	All of the time
a) Has your health restricted you from doing physical activity?	☐ 1	☐ 2	☐ 3	☐ 4	☐ 5
b) Have you felt depressed?	☐ 1	☐ 2	☐ 3	☐ 4	☐ 5
c) Have you felt stressed?	☐ 1	☐ 2	☐ 3	☐ 4	☐ 5

27. **Have you ever been told by a doctor or nurse that you have any of the LONG-TERM health conditions listed below?** (Please only include those conditions that have lasted, or are likely to last, for six (6) months or more.)

Please tick one box for each condition.	Yes	No
a) Arthritis	☐ 1	☐ 2
b) Asthma	☐ 1	☐ 2
c) Any type of cancer	☐ 1	☐ 2
d) Chronic bronchitis or emphysema	☐ 1	☐ 2
e) Diabetes	☐ 1	☐ 2
f) Heart/coronary disease	☐ 1	☐ 2
g) High blood pressure/hypertension	☐ 1	☐ 2
h) Any other serious circulatory condition (e.g. stroke, hardening of the arteries)	☐ 1	☐ 2
i) Other *(please describe)*		

28. In the last year, has a doctor, nurse, or health professional talked to you about physical activity or advised you to do exercise?

Please tick one.

Yes	No
☐ 1	☐ 2

29. Are you:

Male	Female
☐ 1	☐ 2

30. (For women only) Are you pregnant?

No	Yes	How many weeks pregnant?
☐ 1	☐ 2 →	☐

31. **How tall are you without shoes on?** (Please tell us in either centimetres or feet and inches.)

Please check using your driver's licence if you have one.

Centimetres		Feet	Inches
☐	OR	☐	☐

32. **How much do you weigh without your clothes or shoes on?** (Please tell us in either kilograms or stone and pounds.)

Please check using a set of scales if you have them.

Kilograms		Stone	Pounds
☐	OR	☐	☐

33. Which **ONE** of the following best describes your cigarette smoking?

Please tick one.

I smoke daily ☐ 1 → How many cigarettes do you usually smoke each day? ☐

I smoke occasionally ☐ 2

I don't smoke now, but I used to ☐ 3 → What year did you quit smoking? ☐

I have never smoked ☐ 4

Section 7:

YOU AND YOUR HOUSEHOLD

This last section asks a few questions about you and your household. We need to ask these questions as it is important for us to make sure we have a wide variety of people in our study.

34. In which country were you born?

	Australia	Other country, please name
	☐ 1	

35. What is your date of birth (e.g. 23/5/1951)

	Day	Month	Year

36. What is the **highest** educational qualification you have completed?

Tick ONE only.

Year 9 or less		☐ 1
Year 10 (Junior/4th form)		☐ 2
Year 11 (Senior/5th form)		☐ 3
Year 12 (Senior/6th form)		☐ 4
Certificate (trade or business)		☐ 5
Diploma or Associate Degree		☐ 6
Bachelor Degree (Pass or Honours)		☐ 7
Graduate Diploma or Graduate Certificate		☐ 8
Postgraduate degree (Masters degree or Doctorate)		☐ 9
Other *(please describe)*		☐ 10

37. Which **ONE** of the following best describes your current living arrangement?

Please tick one only.

Living alone with no children	☐ 1
Single parent living with one or more children	☐ 2
Single and living with friends or relatives	☐ 3
Couple (married or defacto) living with no children	☐ 4
Couple (married or defacto) living with one or more children	☐ 5
Other *(please specify)* ______	☐ 6

38. How many people in total live in your household?

(Please include yourself, partner, children, and/or anyone else living with you)

☐

39. How many children do you currently have living in your care (either full-time or part-time)?

Please provide the number for each age group.	None	Number aged 0 to 12 months	Number aged 1-5 years	Number aged 6-12 years	Number aged 13-17 years	Number aged 18 years or more
	☐ 1					

40. Do you or someone else in your household own a dog(s)?

Yes	No
☐ 1	☐ 2

41. Do you have a motor vehicle available for your personal use?

Please tick one.	Yes, always	Yes, sometimes	No	Do not drive
	☐ 1	☐ 2	☐ 3	☐ 4

42. On most weekdays (Monday to Friday), which type of transport do you **MAINLY** use to get to and from places?

Please tick the main one.	Public transport	Car or motorcycle	Walk	Bicycle	Other
	☐ 1	☐ 2	☐ 3	☐ 4	☐ 5

43. The next two questions are about walking and cycling for transport. Transport includes things like travel to and from work, to do errands, or to go from place to place. **When answering these questions please do not count walking or cycling for exercise or recreation.**

a) What do you estimate was the total time that you spent walking for transport in the **LAST WEEK**?

Hours ____ *Minutes* ____

If NONE, please write 0

b) What do you estimate was the total time that you spent cycling for transport in the **LAST WEEK**?

Hours ____ *Minutes* ____

If NONE, please write 0

44. Which **ONE** of the following best describes your current employment situation?

Please tick ONE number only.

Full time paid work in a job, business or profession	☐ 1	PLEASE GO TO QUESTION 45 BELOW
Part time paid work in a job, business or profession	☐ 2	
Casual paid work in a job, business or profession	☐ 3	
Work without pay in a family or other business	☐ 4	
Home duties not looking for work	☐ 5	PLEASE GO TO QUESTION 49 ON PAGE 19
Unemployed looking for work	☐ 6	
Retired	☐ 7	
Permanently unable to work	☐ 8	
Student	☐ 9	
Other *(please specify)* ______	☐ 10	

45. This question asks about physical activity in your **MAIN** job. On a usual working day, how often do you do each of the following while you are at work?

Please tick one box for each item.	None of the time	A little of the time	Some of the time	Most of the time	All of the time
a) Standing	☐ 1	☐ 2	☐ 3	☐ 4	☐ 5
b) Walking	☐ 1	☐ 2	☐ 3	☐ 4	☐ 5
c) Heavy labour or physically demanding work	☐ 1	☐ 2	☐ 3	☐ 4	☐ 5

46. About how much time do you spend **SITTING** while at work **ON A USUAL DAY**:

Hours	Minutes
______	______

47. What is your current occupation? (If you have more than one job, we are interested in your main job.)

*Please give full title (for example: Childcare Aide, Maths Teacher, Pastrycook, Commercial Airline Pilot, Apprentice Toolmaker, etc). For **Public Servants**, state official designation and occupation. For **armed services personnel**, state rank and occupation.*

Full title of Occupation: ______

48. In a usual week, how many hours per week do you work in your **MAIN** job?

Numbers of hours

49. What was your **MAIN** occupation when you were 25 years old?

Full title of occupation *(write below)*	Unemployed	Home duties	Other *(please describe)*
	1	2	

50. What was your father's **MAIN** occupation when **you** were 10 years old?

Full title of occupation *(write below)*	Unemployed	Retired	Don't know	Other *(please describe)*
	1	2	3	

51. What was your mother's **MAIN** occupation when **you** were 10 years old?

Full title of occupation *(write below)*	Unemployed	Retired	Home duties	Don't know	Other *(please describe)*
	1	2	3	4	

52. Were you living with both your own mother and your own father when you were 10 years old?

Both parents	Father only	Mother only	Neither parent	Other *(please describe)*
1	2	3	4	

53. Where did you live when you were 10 years old, and when you were 25 years old?

	When you were 10 years old	When you were 25 years old
Country		
State/Territory		
City/Town		
Suburb		
Postcode (if known)		

To help us understand the difficulties that people with different levels of income experience, we would be grateful if you could provide us with an estimate of your total household income.

We know that some people feel uncomfortable providing information about their income, so to help make this easier we have grouped the incomes into broad categories so that your actual household income can't be identified.

Why are we asking about income? An aim of the study is to help make sure that all Brisbane residents, regardless of income, have equal access to the facilities and services they need. By answering this question, you will help us achieve this aim. Please be reassured that your answer will be treated as strictly private and confidential.

54. Please add up the amount of **BEFORE-TAX** income received by **ALL** members of your household, and tick the box that comes closest to this number. Please indicate income either per year, per fortnight, or per week.

Tick one box only.

**Per year**	OR	**Per fortnight**	OR	**Per week**
☐ Less than $15,599		☐ Less than $600		☐ Less than $300
☐ $15,600-20,799		☐ $600-799		☐ $300-399
☐ $20,800-25,999		☐ $800-999		☐ $400-499
☐ $26,000-31,199		☐ $1,000-1,199		☐ $500-599
☐ $31,200-36,399		☐ $1,200-1,399		☐ $600-699
☐ $36,400-41,599		☐ $1,400-1,599		☐ $700-799
☐ $41,600-51,999		☐ $1,600-1,999		☐ $800-999
☐ $52,000-72,799		☐ $2,000-2,799		☐ $1,000-1,399
☐ $72,800-93,599		☐ $2,800-3,599		☐ $1,400-1,799
☐ $93,600-129,999		☐ $3,600-4,999		☐ $1,800-2,499
☐ $130,000 or more		☐ $5,000 or more		☐ $2,500 or more
☐ Don't know				
☐ Don't want to answer this				

FINALLY...

We are planning a follow-up of this study in about 2 years time. We are interested in looking at how changes in your area over this time affect your lifestyle, health and well-being. This valuable information will help create better places for Brisbane residents to live. It would greatly assist us if we were able to contact you again. Please provide as many details as possible below, and make your important contribution to shaping the future of our city.

Your current details			
Name			
Street			
Suburb		Postcode	
Home telephone			
Email address			
Mobile phone			

In case you change address, please provide the contact details of someone **not** living with you who will know where you are if you move (e.g. parent, son/daughter, brother/sister, or close friend).			
Name			
Street			
Suburb		Postcode	
Home telephone			
Mobile phone			

THANK YOU

Thank you for the time and effort you have put into completing this survey for us. It is very much appreciated and the information you have provided will be important for our research.

For confidentiality we will remove this page from the survey

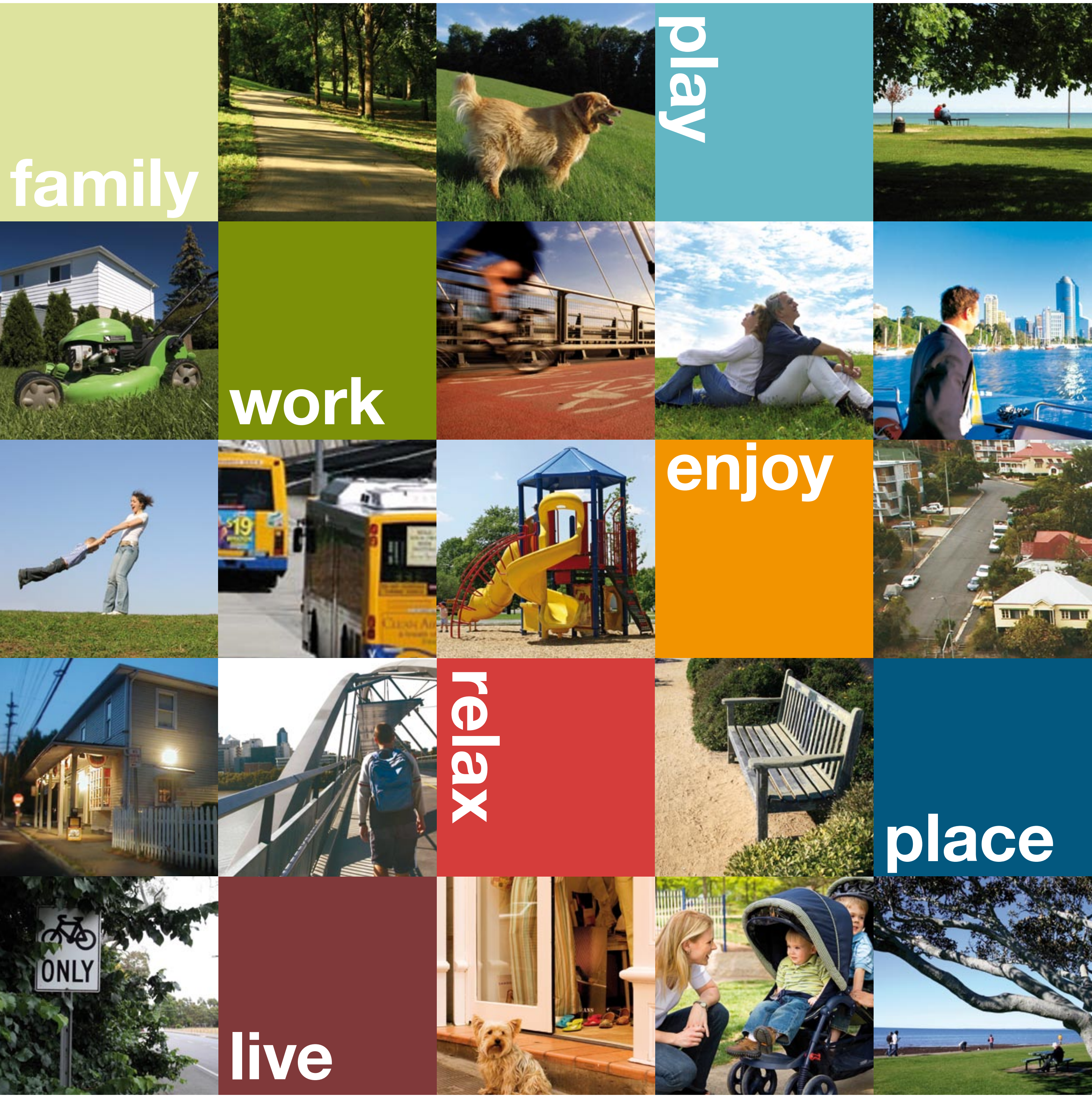

© QUT 2006 Produced by QUT Publications 13452
